# Causes of Sleep Disturbance in Early ASAS Spondyloarthritis: A Retrospective Long-Term Experience

**DOI:** 10.3390/jpm15010031

**Published:** 2025-01-17

**Authors:** Francesca Bandinelli, Andrea Delle Sedie, Ilenia Mallia, Ilaria Mauro, Nikita Pecani, Linda Carli, Lorenzo Esti, Marco Di Carlo, Marina Carotti, Fausto Salaffi

**Affiliations:** 1 Department of Rheumatology, Santa Maria Nuova Hospital, Usl Tuscany Center, 50122 Florence, Italy; 2 Rheumatology Unit, Department of Clinical and Experimental Medicine, University of Pisa, 56126 Pisa, Italy; 3 Rheumatology Unit, Department of Experimental and Clinical Medicine, University of Florence, 50139 Florence, Italy; 4 Rheumatology Unit, Department of Clinical and Molecular Sciences, Università Politecnica of Marche, Carlo Urbani Hospital, Jesi, 60035 Ancona, Italy; 5 Department of Clinical, Special and Dental Sciences, Università Politecnica of Marche, 60126 Ancona, Italy; 6 Radiology Unit, Department of Radiological Science, Università Politecnica of Marche, 60126 Ancona, Italy

**Keywords:** ASAS, early, long term, Spondyloarthritis, sleep disorders

## Abstract

**Introduction:** Sleep disturbance (SD) in the second half of the night due to inflammatory pain was included in the 2009 ASAS classification criteria of Spondyloarthritis (SpA), even though its definition is uncertain. **Aim:** We aimed to investigate SD in early-SpA (e-SpA) patients at T1 (2010–2013), comparing them to long-term SpA (l-SpA) patients at T2 (2023–2024) after at least 10 years of follow-up. **Methods:** At T1, in e-SpA and l-SpA cases, SD, classified as “difficulty in initiating sleep” (DIS), “difficulty in maintaining sleep” (DMS) and “early awakening” (EA), was compared to clinical parameters (ASDAS-CRP, BASDAI, m-HAQ-S, BASMI, MASES, 68/66 joint count, tenderness of sacroiliac joints, fatigue [FACIT] and HADS for anxiety [A] and depression [D]). At T2, e-SpA patients were re-evaluated using the Pittsburgh Sleep Quality Index (PSQI). **Results:** At T1, 45% of 166 SpA patients had SD; in e-SpA patients (60), SD correlated with sacroiliac pain (DMS) BASDAI, FACIT and HADS-D (EA); in l-SpA patients (106), it correlated with HADS-A (DIS), BASDAI and FACIT (DMS). At T2, e-SpA patients showed a high PSQI in 51.5% of cases, correlated with T2-ASDAS-CRP and T2-BASDAI. Moreover, T1-ASDAS-CRP was predictive of T2-PSQI. **Conclusions:** SD is more specific for inflammatory pain in e-SpA and might be influenced by disease activity also in long-term disease.

## 1. Introduction

In recent years, we observed an increased trend of nocturnal sleep deprivation in modern societies having a deep impact on physical and emotional health and on brain homeostasis [1].

The benefits of sleep-dependent human functions have been widely demonstrated in terms of learning capacity and memory consolidation. Single sleep phase abnormalities, in particular rapid eye movement (REM) and slow wave deep sleep (N3), and the change of their harmonic sequence, might cause procedural and declarative memory disturbances [1].

Sleep disorders (SD) are gender- and age-related, might change during life following brain hormonal and structural modifications [2,3] and might be influenced by multifaceted interaction of neurological, dysmetabolic, respiratory and psychiatric disorders [4]. The recent COVID-19 pandemic has also increased the level of stress in terms of coping, psychological disorders and SD, with important consequences in rheumatic diseases [5]. Indeed, sleep deprivation might impair “resilience”, with a progressive loss of disease control and impulse vigilance [1,6]. This deterioration is attributed to a suppression of the prefrontal cortex and temporal lobes, which are believed to play an important role in processing affect and emotions, causing a disequilibrium of the sleep–awake circadian alternance [1,6].

Some recent evidence indicated that the neuronal network of sleep homeostasis might influence the immunity response against infections and inflammatory diseases [7], even if the relationship between insomnia and immune deregulation is still uncertain [2].

In a rat model, after spinal cord transection, increased expression of tumor necrosis factor-alpha (TNF-α) and interleukin-1 (IL-1) was found in apoptotic neurons of the pontine reticular formation [8]; thus, in inflammatory diseases, long-term plastic brain deformation was also hypothesized as a potential cause of progressive decline of biological functions, such as sleep maintenance, behavior, appetite and mental status [6,7,9].

In particular, reticular area abnormalities might exert a powerful modulation of microglia, to release cytokines and chemokines with proinflammatory consequences on SD and cognitive functions through neuronal ascending fibers [10].

In previous studies, SD was often only described as a possible cause of fatigue in Spondyloarthritis (SpA) patients [11], but only recently has it has been considered as a treatment outcome in clinical trials [12,13].

In SpA patients, SD prevalence varies in the literature from 15.4% to 80%, according to nonuniform clinical definitions (i.e., insufficient resting sleep or difficulty of waking up, mostly due to back pain) [14].

After the publication of ASAS criteria in 2009 [15], the Berlin principles, accompanying a description of inflammatory pain, described insomnia in the second half of the night as a major criterion for diagnosis, even though its meaning was not clearly defined in successive editorials and articles.

The first decade dramatically influences the natural and radiological course of the disease [16], even if the diagnosis might be delayed by gender, social and genetic characteristics of patients [17,18]. Furthermore, in long-term SpA (l-SpA) the progression of articular impairment and comorbidities (respiratory disorders, weight gain and depression) [19] might influence quality of sleep and predispose to obstructive apnea [14], which is a possible confounding variable for Berlin ASAS criteria application.

In the present study, we aimed to retrospectively evaluate SD in SpA after 2009, comparing early SpA (e-SpA) to l-SpA (>2 years since the onset of symptoms). As a second outcome, we explored SD, in the same population of e-SpA after at least 10 years of routine follow-up, to correlate SD with disease activity indices.

## 2. Materials and Methods

### 2.1. Study Design

We conducted a retrospective analysis (n.2015/0016289, Florence Ethical Committee), of Italian SpA patients referred to the out-patient clinics of the USL Tuscany Center, University of Florence and University of Pisa, between 2010 and 2013 (T1), by means of a complete clinical examination, sleep disorder evaluation and questionnaires usually included in daily clinical practice; we excluded those with important comorbidities at baseline (i.e., severe pneumopathy and cardiopathy, brain disorders and trauma, alcohol and substance abuse and a definitive diagnosis of fibromyalgia), that might have an impact on SD.

### 2.2. Participants

Data on SpA patients who visited between 2010 and 2013, fulfilling ASAS criteria [15] and aged over 18 years old, were retrospectively examined by means of document folders and electronic charts, and patients with a complete routine clinical examination and questionnaires at T1 were selected.

Successively, e-SpA (defined as symptom duration at T1 shorter than 2 years [20]) patients, not lost at follow-up for the next 10 years, were re-evaluated between July 2023 and July 2024 (T2), using their medical files and by teleconsultations in case of doubt, to exclude death, alcohol and substance abuse, severe comorbidities (heart, lung, infections, cancer, infection with hospitalization, psychosis and brain trauma) and definitive diagnosis of fibromyalgia. Once they were selected, SD questionnaire and disease activity scores were obtained in face-to-face or telemedicine interviews.

Patients initially sent by general practitioners were followed according to regional flowchart guidelines and Italian Health Ministry legislation (law of 22 April 2021, no. 52, valid until the repeal of the law of 10 August 2023, no. 105). Privacy and informed consent (for anonymous analysis and publication of routine clinical data) were saved in the Argos electronic chart of the USL Tuscany Center, as per the Declaration of Helsinki on investigation involving humans and according to the Tuscany Region Institutional Review Board resolution (No. 450) and Italian legislation (authorization No. 9, law of 12 December 2013).

### 2.3. Measurements

Data on demographic and serological biomarkers, rheumatological and general clinical conditions, social factors (job and family), alcohol and drugs addiction and therapy were collected from patient records in the multidisciplinary Argos and Pleiades electronic charts (of the USL Tuscany Center and University of Pisa, respectively) and completed through face-to-face and telemedicine interviews, when necessary.

At T1, SD of e-SpA and l-SpA patients was described using the international definition of insomnia according to the DSM-5 criteria: SD in the first part of the night was defined as “difficulty in initiating sleep” (DIS), while in the second part, SD was shared between “difficulty in maintaining sleep” (DMS) and “early awakening” (EA) [21].

Data on clinical evaluations following the ASAS guidelines [15] were also collected: BASMI for the spine (0–13), MASES for entheses (0–9), and tender (0–68) and swollen (0–66) counts for joints. Sacroiliac tenderness (0–2) was routinely evaluated by sacral sulcus palpation immediately medial to the posterior superior iliac spine and by provocative tests for joint pain: (a) distraction test, (b) lateral compression test, (c) sacroiliac thrust test and (d) Faber test [22].

Questionnaires completed at face-to-face interviews on disease activity were considered: the Bath Ankylosing Spondylitis Disease Activity Index (BASDAI) and Axial SpA Disease Activity Score (ASDAS) [15,23]. Furthermore, the Hospital Anxiety and Depression Scale (HADS) [24], Functional Assessment of Chronic Illness Therapy (FACIT) and modified health-related quality of life questionnaire for SpA (m-HAQ-S) were also analyzed [25].

BASDAI (0–10) was composed of six questions about the five major symptoms of SpA: fatigue, spinal pain, peripheral joint pain or swelling, tenderness and morning stiffness. The questions were arranged according to a VAS scale with “none” (0) to “very severe” (10) labels for all parameters except morning stiffness duration, which was measured as “0” until “2 or more hours”. Average scores from questions 5 and 6 for stiffness are added to the other scores from questions 1 to 4, and the total sum is divided by 5 to provide the final score.

ASDAS-CRP was calculated on C-reactive protein expressed in mg/dL using the following formula: 0.1216 × total back pain + 0.1106 × patient global + 0.0736 × peripheral pain/swelling + 0.0586 × duration of morning stiffness + 0.5796 ln (CRP + 1), and was preferred to ASDAS-ESR.

The HADS was considered as a routine self-report rating scale (0–21 range) of 14 items on a 4-point Likert scale (range 0–3), designed to measure anxiety (HADS-A) and depression (HADS-D) (7 for each subscale) and to guide further psychiatric examination. The total score is the sum of the 14 items, and for each subscale the score is the sum of the seven respective items. Insomnia was not included as a somatic aspect of depression in this score.

The FACIT score is a health-related quality of life questionnaire targeted at the management of chronic illness and containing 13 measures to assess self-reported fatigue (Likert scale from 4 = not fatigued to 0 = very fatigued) and its impact upon daily activities and function.

The m-HAQ-S scale is adapted from a previous HAQ generic questionnaire and contains 20 items on dressing, arising, gripping, eating, bathing and walking ability contained in the original HAQ, and five specific questions about neck function and static resistance (driving a car, using the rearview mirror, carrying heavy shopping bags, sitting in the same position for long periods and working at desk). The final score for each item ranges from 0 (unable to do) to 3.

The history of e-SpA patients during almost ten years of disease (T2) was retrospectively considered from medical files, selecting subjects not lost at follow-up and without exclusion criteria.

The e-SpA patients included were successively re-evaluated by face-to-face or telemedicine interviews between July 2023 and July 2024.

At T2, SD was measured using the Italian version of the Pittsburgh Sleep Quality Index questionnaire (PSQI) [14,26,27], and disease activity was scored using ASDAS-CRP and BASDAI.

The PSQI questionnaire, created as psychometric analysis instrument, was initially based on a combination of Likert-type and open-ended questions, then converted to scaled scores using provided guidelines. Respondents are asked to indicate how frequently they have experienced certain sleep difficulties over the past month and to rate their overall sleep quality. The scale consists of 19 items and measures seven components of SD: subjective sleep quality, sleep latency, sleep duration, habitual sleep efficiency, sleep disturbances, use of sleep medication and daytime dysfunction. Five additional questions rated by the respondent’s roommate or bed partner are also included. Scores for each question range from 0 to 3, with higher scores indicating more severe SD. A global PSQI score is calculated, corresponding to the sum of the individual scores of the seven components (0–21). A PSQI global score higher than five is considered the cut-off between “good sleepers” (<5) and “poor sleepers” (>5).

### 2.4. Statistical Analysis

The sample size of the population, based on a similar study on SD in 150 SpA patients [14], gained >80% power with an α level of 0.05 and β of 0.2. The Kolmogorov–Smirnov and Shapiro–Wilk tests were used to evaluate the distribution of variables. Descriptive statistics were expressed as mean, standard error of the mean (SEM) and confidence interval (CI) for continuous parameters and as a percentage for categorical variables, as appropriate. Categorical data were compared using the Fisher exact test; single continuous parameters (i.e., age) were compared using the Mann–Whitney test, as appropriate. At T1, SD in different parts of the night was analyzed in comparison with clinical parameters with multi-parametric linear regression. At 10 years follow-up of e-SpA patients, T2-PSQI was compared to T2-BASDAI and T2-ASDAS-CRP using Pearson’s r test and to T1-BASDAI and T1-ASDAS (to establish predictivity) using simple linear regression. The level of statistical significance was set at *p*-value ≤ 0.05. All statistical analyses were performed using GraphPad prism 8.0 and reported in conformity with STROBE guidelines.

## 3. Results

**Figure 1:**
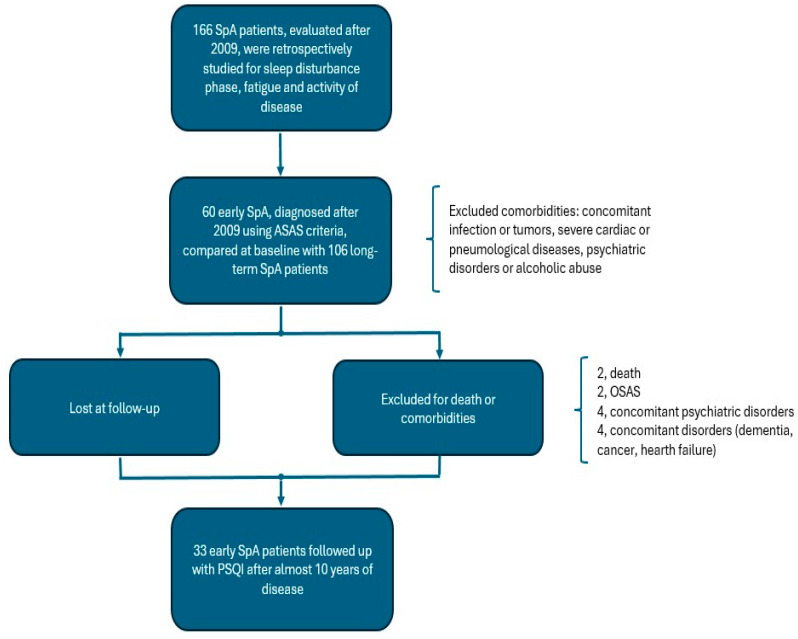
Flow chart of the T1 baseline (2010–2013) clinical assessment of the ASAS SpA population and T2 (after almost 10 years) follow-up of e-SpA patients using the Pittsburgh Sleep Quality Index (PSQI).

Figure 1 shows the flow chart of the study. At T1, 166 SpA patients were included and classified as 60 e-SpA and 106 l-SpA, based on recent ASAS guidelines [20].

At T2, the clinical data of 45 e-SpA, not lost at 10 years of follow-up, were studied: 2 had died, 4 were excluded due to psychiatric problems (3 were diagnosed with psychotic disorders and 1 with major depressive disorder) and 4 for other severe chronic diseases (1 breast cancer, 1 heart failure and 2 dementia). From the 35 e-SpA patients included and investigated with PSQI, two patients were selected for polysomnography examination and excluded for nocturnal apnea diagnosis. Finally, 33 e-SpA patients without comorbidities were selected for T2-PSQI and T2-disease activity evaluation. No patients included were hospitalized for COVID-19 infection or were obese (BMI > 30) during follow-up.

### 3.1. T1 in e-SpA and l-SpA: Comparison of SD

At T1, the demographic characteristics were similar in e-SpA and l-SpA (Table 1). SpA patients were naïve for chronic steroid treatment and were not included in specific protocols for biological disease-modifying antirheumatic drugs (b-DMARDs). l-SpA patients were treated more frequently with b-DMARDs than e-SpA (Table 1). The values of other clinical parameters of e-SpA and l-SpA patients at T1 are shown in Supplementary Table S1.

As shown in Table 2, 75/166 patients (45%) (29/60 e-SpA, 48.3%; 46/106 l-SpA, 43.4%) claimed SD, with a mild, not significant, higher prevalence of early awakening (EA) in l-SpA and difficulty in maintaining sleep (DMS) in e-SpA patients.

In a multiple linear regression test (Table 2), in l-SpA patients, difficulty of initiating sleep (DIS) was correlated with HADS-A, and DMS with BASDAI and FACIT. On the other hand, in e-SpA patients, DMS was correlated with sacroiliac tenderness, and EA with BASDAI, FACIT and HADS-D.

### 3.2. T2 of the e-SpA Population: PSQI of e-SpA Patients at T2 and Its Correlation with Disease Activity Scores

After ten years of follow-up (T2), the social factors of 33 selected e-SpA patients were weighted: five patients lost their jobs, while three gained new work; two divorced and two married.

At T2, most patients showed low disease activity scores: BASDAI < 4/10 in 27/33 (81.8%) and ASDAS-CRP < 2.1 in 22/33 (66.6%).

8/33 (24.2%) were treated with b-DMARDs (4/33 [12.1%], in monotherapy, and 13/33 (36.3%) with s-DMARD treatments); 16/33 (48.5%) were discontinued from all DMARDs.

17/33 (51.5%) of e-SpA patients were poor sleepers (the T2-PSQI score was higher than 5). The T2-PSQI (5.7 ± 0.6, 4.3–7 CI) correlated with disease activity indices: T2-BASDAI (2.1 ± 0.3, 1.5–2.7 CI) *p* < 0.0001 and T2-ASDAS-CRP (1.8 ± 0.2, 1.4–2.1 CI) *p* < 0.0008 (Spearman’s r test). On the other hand, T2-PSQI was independent of DMARD use: the T2-PSQI did not significantly differ (Mann–Whitney non-parametric unpaired test) between patients discharged (6.1 ± 0.9, 4.2–8) and those still on treatment (4.8 ± 0.8, 3–6.7).

### 3.3. Predictivity of e-SpA Activity Scores at T1 to T2-PSQI

**Figure 2:**
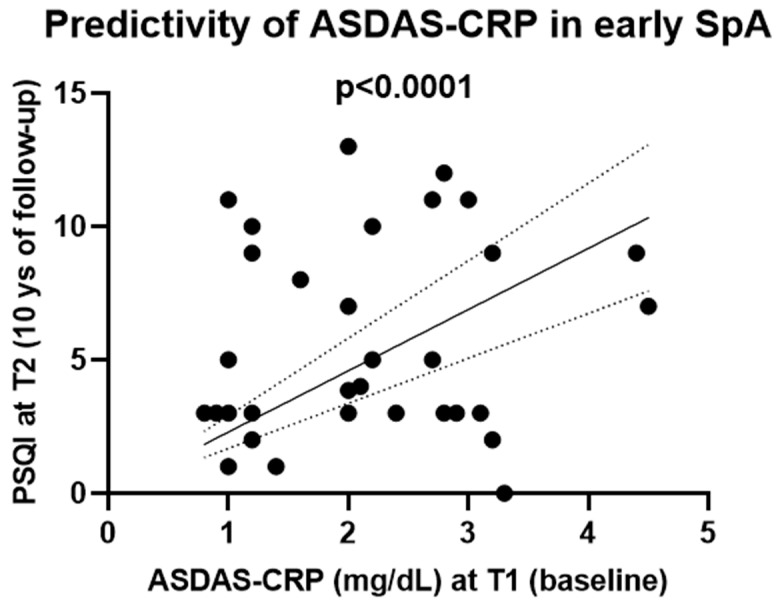
Predictivity of e-SpA disease activity score (ASDAS-CRP, mg/dL) at T1 to Pittsburgh Sleep Quality Index (PSQI) at T2 follow-up.

Among activity indices at T1, T1-ASDAS-CRP (2.3 ± 0.1, 2–2.6 CI) and T1-BASDAI (3.2 ± 0.3, 2.5–3.9 CI), only T1-ASDAS-CRP was predictive (Figure 2) to T2-PSQI at follow-up (*p* < 0.0001), (simple linear regression).

## 4. Discussion

Our results showed that SD was present in almost 50% of SpA patients and persisted, in e-SpA patients 10 years after diagnosis, independently of treatments or social factors, according to new evidence about SpA [4,28,29,30,31,32,33,34].

Following the latest ASAS definition, e-SpA, of less than two years of duration, not influenced by radiological staging [20,35,36], showed, in the second part of the night, a DMS specifically correlated with sacroiliac pain. On the other hand, EA and DIS in e-SpA and l-SpA patients, respectively, might be conditioned by depression and anxiety.

Thus, only in e-SpA patients, SD seemed more prone to relate to inflammatory pain according to the Berlin definition contained in the ASAS criteria [15].

While mood disorders seemed to have a similar distribution in different stages of the disease and not be conditioned by handicap severity [37], the repetitive and persistent awakenings [27] were suspected to have an impact on N3 and REM homeostasis, with longstanding consequences in terms of resilience, fatigue and cognitive debriefing [9,38].

On the other hand, in early phases, microglia activation might also be triggered by inflammatory mechanisms, that play an important role in mood disorder onset [39,40,41].

L-SpA might also be conditioned by progressive distortion of body image and professional and relational problems [42]

A total of 36% of SpA patients interviewed in the latest large survey experienced important limitations in career development and perspectives; 15% were unemployed (only 4% as a direct consequence of the disease), and 50% were handicapped [43].

Even if our study was limited by the smaller cohort studied, SD seemed independent of family and job conditions, due probably to the good disease control of early treatment, which is correlated with higher work stability, as confirmed by other larger international studies [44].

Furthermore, e-SpA patients showed a high PSQI score in more than half of patients at T2 (10 years of disease), which correlated with disease activity. T1-ASDAS-CRP was also predictive of T2-PSQI.

Even if our study might show limitations due to its retrospective nature and long-term observation period with possible patient loss at follow-up, the interest of our first exploratory study is to offer a first point of view on a complex “iceberg” clinical challenge, not always well understood in routine practice, and to inspire future research on larger cohorts.

As far as we know, based on HLA-B27, IL23R, ERAP1 and IL-1 genetic predisposition, TNF-α and IL-1 have an influence on glial cells of the central nervous system [45], which might persist in different phases of the disease in the long term.

The literature highlighted an association between sleep deprivation or depression and high levels of specific cytokines, (IL-1, IL-6 and TNF-α) [10,46]. On the other hand, the duration and quality of sleep might impair innate immunity [7] and influence monocytes and lymphocytes that usually release IL-6 and TNF-α [10], resulting in a complex vicious cycle.

A few controlled studies on anti-TNF-alpha [4], ixekizumab [13] and bimekizumab [47] showed significant improvement in sleep quality in SpA patients during therapy when compared to a placebo. Moreover, thalidomide, used for its immunosuppressive action on IL-1, IL-6 and TNF-α in patients refractory to other treatments, was considered effective on PSQI [48].

Unfortunately, until now, the specific correlation of cytokine levels with polysomnography results has not been investigated in SpA patients.

We know from animal models that inflammatory cytokines, through the disrupted blood–spinal barrier [8,49,50] lead to massive release of amino acid-like glutamate that activates the calcium channel, with consequent neuronal apoptosis [8,49]. In long-term disease, a progressive plastic remodeling of the reticular system might be hypothesized in SpA patients by the two main apoptotic pathways: the extrinsic (which leads to neuronal death by caspase) and the intrinsic (by mitochondrial activation) [8,49].

Even if the role of neuroinflammation was not greatly demonstrated in e-SpA and l-SpA patients and the effect of cytokines on reticular formation is still under debate, this fascinating modern research area might be supported by future more specific studies on humans and animals.

## 5. Conclusions

SD represents a great limitation on the quality of life of SpA patients, and is often underestimated and investigated only at first diagnosis.

According to our results, SpA patients frequently complained of SD, and, in the second part of the night, it seemed to be specifically linked to sacroiliac pain only in early disease.

Furthermore, interestingly, SD correlated with disease activity, both in e-SpA and l-SpA patients, even if it was also conditioned by mood disorders and fatigue.

Moreover, ASDAS-CRP at T1 seemed to be predictive of SD at T2, and persists after ten years of disease in e-SpA patients.

This study presents some limitations due to its retrospective nature and relatively low number of patients enrolled; however, given the interesting results obtained, we hope that future larger studies will be performed to shed light on this important topic.

Indeed, we are convinced that the grey area of a ten-year “window of opportunity” remains at the forefront of the modern approach to the disease, and we suppose that the road to knowledge of the definition of neuroinflammatory changes of the central nervous system during the different phases of SpA is still to be achieved.

A multidisciplinary approach focused on basic (cytokine) and clinical (polysomnography) research is deemed essential to better manage this misunderstood aspect of the disease.

## Figures and Tables

**Table 1:** Demographic and treatment data of long-term (l-SpA) and early (e-SpA) Spondyloarthritis patients at T1.

Parameters	l-SpA (n. 106)	e-SpA (n. 60)	*p* Significance
F/M ratio	42:64	37:23	Ns (*)
Age	55.3 ± 1.3 (52.6–58)	51 ± (47.7–54.3)	Ns (**)
b-DMARDs	**33/106 (31%)**	5/60 (8.3%)	***p*** 0.009 (*)
s-DMARDs	41/106 (38.6%)	**34/60 (56.6%)**	***p*** 0.003 (*)

All parameters are expressed in mean ± standard error of the mean (SEM) and confidence interval (CI); *p* significances of Fisher (*) and Mann–Whitney tests (**) were considered lower than 0.05. Abbreviations: b-DMARDs: biological disease-modifying antirheumatic drugs; e-SpA: early spondyloarthritis; F: female; l-SpA: long-term Spondyloarthritis; M: male; Ns: nonsignificant; s-DMARDs: synthetic disease-modifying antirheumatic drugs.

**Table 2:** Comparison of insomnia during different parts of the night to clinical examination and questionnaires in long-term (l-SpA) and early (e-SpA) Spondyloarthritis at T1.

l-SpA Baseline	Part of Night	Parameters	*p* Significance
13.2% (Fisher test, ns *)	DIS	**HADS-A (7.2 ± 0.6, 5.9–8.4)** Other parameters	0.03 (**) Ns
33% (ns *)	DMS	**BASDAI (3.2 ± 0.3, 2.5–3.8)** **FACIT (14.6 ± 1.6, 11.3–17.9)** Other parameters	0.01 (**) 0.003 (**) Ns
24.5% (ns *)	EA	All parameters	Ns
**e-SpA Baseline**	**Part of Night**	**Parameters**	***p* Significance**
15% *	DIS	All parameters	Ns
40% *	DMS	**Sacroiliac tenderness** **(0.5 ± 0.1, 0.3–0.7)** Other parameters	0.03 (**) Ns
16.6% *	EA	**BASDAI (3.2 ± 0.3, 2.5–3.8)** **HADS-D (5.7 ± 0.5, 4.6–6.8)** **FACIT (14.6 ± 1.6, 4.6–6.8)** Other parameters	0.04 (**) 0.02 (**) 0.007 (**) Ns

All parameters are expressed in mean ± standard error of the mean (SEM) and confidence interval (CI); *p* significances of Fisher (*) and Mann–Whitney tests (**) were considered lower than 0.05. Abbreviations: BASDAI: Ankylosing Spondylitis Disease Activity Index; DIS: difficulty in initiating sleep; DMS: difficulty in maintaining sleep; EA: early awakening; e-SpA: early spondyloarthritis; FACIT: Functional Assessment of Chronic Illness Therapy—Fatigue Scale; HADS: Hospital Anxiety (A) and Depression (D) Scale; l-SpA: long-term Spondyloarthritis; Ns: not significant.

## Data Availability

The data are available, if requested.

## Supplementary Materials

The following supporting information can be downloaded at https://www.mdpi.com/article/10.3390/jpm15010031/s1: Table S1: Data of baseline parameters present in early spondyloarthritis (e-SpA) and long-term disease (l-SpA).
