# Supplementary material for: Causes of Sleep Disturbance in Early ASAS Spondyloarthritis: A Retrospective Long-Term Experience
Source: J Pers Med. 2025 Jan 17;15(1):31. doi: 10.3390/jpm15010031 (PMC11767170; doi:10.3390/jpm15010031)
Supplement: Supplementary file 1 [file jpm-15-00031-s001.zip › jpm-3337738-supplementary.pdf]

| Baseline parameters | e-SpA baseline (n. 60) | l-SpA (n.106) |
|---|---|---|
| BASDAI | 3.6±0.3 | 3.9±0.2 |
| ASDAS-CRP | 2.3±0.1 | 2.4±0.1 |
| HADS-A | 7.2±0.6 | 6.8±0.4 |
| HADS-D | 5.7±0.5 | 6.1±0.3 |
| FACIT | 14.6±1.6 | 16.1±1.1 |
| m-HAQ-S | 0.6±0.1 | 0.7±0.1 |
| Tender joints count (0-68) | 1.9±0.5 | 2±0.4 |
| Swollen joints count (0-68) | 0.7±0.1 | 0.8±0.2 |
| MASES | 2.6±0.5 | 1.2±0.2 |
| BASMI | 0.8±0.1 | 1.5±0.2 |
| Sacroiliac tenderness | 0.6±0.1 | 0.3±0.06 |

**Legend:** All parameters are expressed in mean ± standard error mean (SEM) and confidence interval (CI); **Abbreviations:** ASDAS-CRP: Axial Spondyloarthritis Disease Activity Score calculated on C protein active (mg/dL); BASDAI: Ankylosing Spondylitis Disease Activity Index; BASMI: Bath Ankylosing Spondylitis Metrology Index; e-SpA: Early Spondyloarthritis; FACIT: Functional Assessment of Chronic Illness Therapy – Fatigue Scale; HADS: Hospital Anxiety (-A) and Depression (-D) Scale; l-SpA: long-term Spondyloarthritis; MASES: Maastrich Ankylosing Spondylitis Enthesitis Score ; m-HAQ-S: health assessment questionnaire modified for SpA

**Table S1. Data of baseline parameters present in early Spondyloarthritis (e-SpA) and long-term disease (l-SpA)**
